# Artificial Intelligence-Assisted Meta-Analysis of the Frequency of ACE I/D Polymorphisms in Centenarians and Other Long-Lived Individuals

**DOI:** 10.3390/ijms24043411

**Published:** 2023-02-08

**Authors:** Lingxuan Li, Shin Murakami

**Affiliations:** Department of Basic Sciences, College of Osteopathic Medicine, Touro University California, Vallejo, CA 94592, USA

**Keywords:** age-related comorbidity, angiotensin-converting enzyme, centenarians, meta-analysis, longevity, life extension

## Abstract

Current research on the angiotensin-converting-enzyme (*ACE*) gene has yielded controversial results on whether different *ACE* polymorphisms are linked with human longevity. *ACE* polymorphisms are a risk factor for Alzheimer’s disease and age-onset diseases that may contribute to the mortality of older people. Our goal is to consolidate existing studies, using artificial intelligence-assisted software to come to a more precise understanding of the role of the *ACE* gene in human longevity. The I (insertion) and D (deletion) polymorphisms in the intron are correlated with the levels of circulating ACE; homozygous D (DD) is high, and homozygous I (II) is low. Here, we performed a detailed meta-analysis of the I and D polymorphisms using centenarians (100+ years old), long-lived subjects (85+ years old), and control groups. *ACE* genotype distribution was analyzed across a total of 2054 centenarians and 12,074 controls, as well as 1367 long-lived subjects between the ages of 85–99, using the inverse variance and random effects methods. The *ACE* DD genotype was found to be favored in centenarians (OR: 1.41 (95% CI: 1.19–1.67), *p* < 0.0001) with a heterogeneity of 32%, and the II genotype slightly favored the control groups (OR: 0.81 (95% CI: 0.66–0.98), *p* = 0.03) with a heterogeneity of 28%, corroborating results from previous meta-analyses. Novel to our meta-analysis, the ID genotype was found to be favored in control groups (OR: 0.86 (95% CI: 0.76–0.97), *p* = 0.01) with a heterogeneity of 0%. The long-lived group showed a similar positive association between the DD genotype and longevity (OR: 1.34 (95% CI: 1.21–1.48), *p* < 0.0001) and a negative association between the II genotype and longevity (OR: 0.79 (95% CI: 0.70–0.88), *p* < 0.0001). The long-lived ID genotype did not show significant findings (OR: 0.93 (95% CI: 0.84–1.02), *p* = 0.79). In conclusion, the results suggest a significant positive association of the DD genotype with human longevity. However, despite the previous study, the results do not confirm a positive association of the ID genotype with human longevity. We suggest a few important paradoxical implications: (1) inhibition of ACE can increase longevity in model systems from nematodes to mammals, seemingly opposite to the finding in humans; (2) exceptional longevity associated with homozygous DD is also associated with age-related diseases with higher mortality risks in homozygous DD. We discuss *ACE*, longevity, and age-related diseases.

## 1. Introduction

Human life expectancy has increased in the past millennia since the studies of longevity in ancient Greek and Roman populations [1,2,3]. The study of aging, including the biology of aging and populations of older people, has suggested that longevity is influenced by a combination of genetic, lifestyle, and environmental factors [4]. The search for genetic causes of longevity has implicated several possible candidate genes, including *SIRT1*, *APOE*, *FOXO3A*, *ACE*, *ATM*, *NOS1*, *NOS2*, *KLOTHO*, and *IL6* [5,6]. Of these, *ACE* encodes an angiotensin-converting enzyme, which has two functions with opposing effects on health [7,8]. First, ACE is an essential enzyme for hemodynamic control via the renin-angiotensin-aldosterone system and the kinin-kallikrein system. ACE converts the inactive angiotensin I to the active angiotensin II, a potent vasoconstrictor, and inactivates bradykinin, a potent vasodilator. ACE inhibitors are one of the first-line treatments for hypertension and congestive heart failure. In this function, ACE inhibition is beneficial to health through controlling hypertension. Secondly, ACE is an amyloid-degrading enzyme that can reduce the proteo-toxicity of amyloid. Importantly, *ACE*, when altered, is a risk factor gene in Alzheimer’s disease [9,10]. *ACE* alterations are associated with Alzheimer’s disease [8]. In this function, ACE inhibition may be deleterious to the health through amyloid toxicity. Thus, the effect of *ACE* on longevity may differ depending on the health conditions of advanced aging [8]. Thus, we reason that long-lived groups should be included in this study.

The *ACE* gene is located on chromosome 17q23. The *ACE* I/D polymorphisms were first identified in 1990; they are characterized by the presence (insertion, I) or absence (deletion, D) of a 287-bp (base pair) Alu repeat sequence in intron 16 [11]. The I/D polymorphisms are correlated with the serum levels of circulating *ACE*; the D-allele is higher, and the I-allele is lower [12]. DD individuals have approximately twice the plasma ACE levels as II individuals do [11,13]. Other meta-analyses have also shown a positive association between the D-allele and increased risk of essential hypertension [14], ischemic stroke [15], coronary artery disease [16], left ventricular hypertrophy [17], and pneumonia [18]. In addition, the D-allele has been linked with higher mortality rates from COVID-19 [19].

Paradoxically, though the *ACE* D allele is associated with the risk of cardiovascular disease (CVD), hypertension, Alzheimer’s disease, and other common causes of death in older people, a previous study noted an increased frequency of the DD genotype among a group of centenarians from France [20]. Since then, centenarian studies have been performed across different cohorts, resulting in conflicting results. A more recent study suggests that the DD genotype and the D allele are enriched in centenarians [21], whereas another study suggests that there is no significant difference among the I and D alleles [22,23]. In this meta-analysis, we further consolidated these findings to more precisely determine whether the D polymorphism is enriched in centenarians and long-lived individuals.

## 2. Results

### 2.1. AI-Assisted Software and Centenarian Results

We tested the following AI-assisted programs for meta-analysis: ASReview, Colandr, Parsifal, and Rayyan. Of the programs, Colandr was selected for its accessibility and intuitive user interface. It is assisted by machine learning and natural language processing, which provide an integrated data extraction function and seven additional functions (set-up review, piloting or scoping, literature search, duplicate check, article screen, data coding, appraisal, and documentation) [24]. It uses machine-learning algorithms to determine the relevance of a given study based on other studies deemed relevant by the researcher and orders the list of unscreened studies based on calculated relevance, though the final decision to include or exclude a study requires a critical appraisal by the researcher. Compared with other software we tested, Colandr had the quickest machine-learning algorithm, providing feedback on study relevance after approximately five studies marked for inclusion by the researcher, compared with eight or more studies for other programs. Overall, Colandr provides a good framework for the systematic review of the literature and offers helpful feedback to expedite the process.

**Figure 1:**
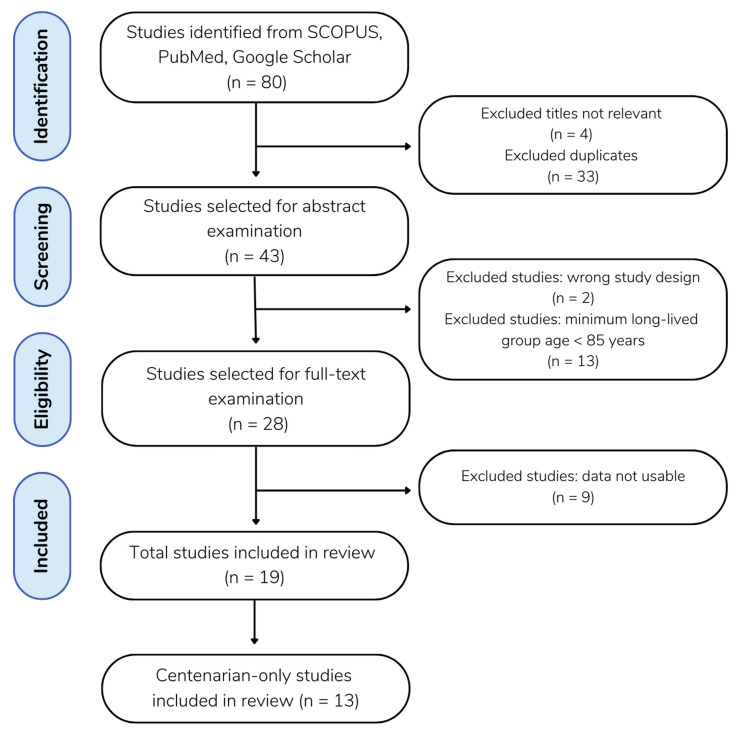
Flow diagram of the literature search. A total of 80 studies were gathered from SCOPUS (n = 39), PubMed (n = 26), and Google Scholar (n = 15). Excluding 33 duplicates and 4 studies with irrelevant titles, 43 remaining studies were selected for abstract examination. Following the abstract screening, 2 studies were excluded for having the wrong study design, and 13 studies were excluded for defining their minimum long-lived population age to be < 85 years of age. A full-text review was performed on the remaining 28 studies, and 9 of those were excluded, as the data was not usable, meaning that the study collected and presented data in a form other than the frequency of individuals’ genotypes, and that the frequency of genotypes was not able to be calculated from the data available.

Our literature search is summarized in Figure 1. The extracted data of centenarians from the identified studies are summarized in Table 1. The meta-analysis included 13 studies [20,22,25,26,27,28,29,30,31,32,33,34,35] involving a total of 2054 centenarians and 10,986 controls. Ethnicities of the studied populations were Caucasian (n = 10), Korean (n = 1), Uyghur (n = 1), and Han Chinese (n = 1). This meta-analysis investigated the age groups that could be analyzed. We found three age groups that consistently showed up among the previous studies, resulting in centenarians (100+ years old), long-lived groups (85+ years old), and control groups. The range of the control groups was diverse among previous studies. We set the ages of control groups as 18–85 years old, which was judged to be inclusive of most of the studies.

The major differences from the previous meta-analysis were as follows. First, we used different age groups for a more precise understanding of the effect of *ACE* on longevity. The previous meta-analysis compared the frequencies of various combinations of I and D (i.e., DD vs. II; DD vs. ID; ID vs. II; DD + ID vs. II; and DD vs. ID + II) [21] and lacked comparisons among different age groups. Secondly, we excluded a study [36] that was used in the previous meta-analysis [21] for the following reason: the excluded study used 62–88 years old, which overlapped with the age range for the long-lived group (85+ years old) commonly used in previous studies (see II. Long-Lived Results). Finally, we incorporated AI-assisted software to reduce the burden of the meta-analysis studies.

**Figure 2:**
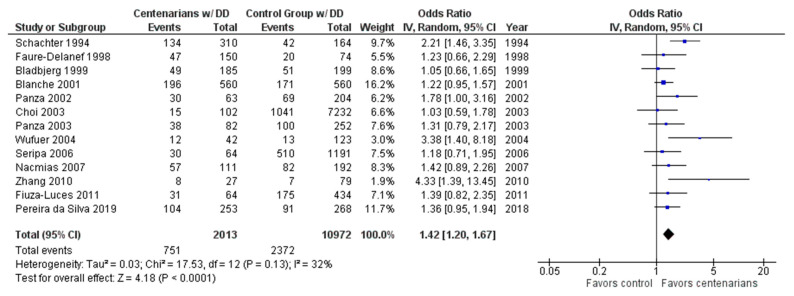
Comparison of DD genotype frequency in centenarians and control groups. The ages of centenarians and control groups are as described in Table 1. The *p*-value is indicated by P in the Figure.

As shown in Figure 2, the association between the DD genotype and exceptional longevity was analyzed in 13 studies [20,22,25,26,27,28,29,30,31,32,33,34,35]. The DD genotype was more frequent among centenarians than in controls, with an OR of 1.41 (95% CI: 1.19–1.67, *p* < 0.0001) with mild heterogeneity (32%), indicating a significant positive association between the DD genotype and longevity.

**Figure 3:**
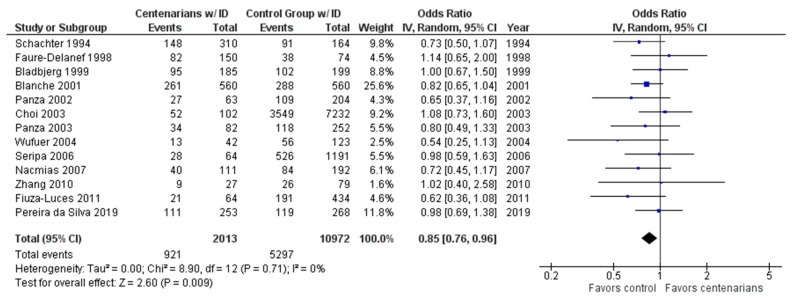
Comparison of ID genotype frequency in centenarians and control groups. The ages of centenarians and control groups are described in Table 1. The *p*-value is indicated by P in the Figure.

As shown in Figure 3, the association between the ID genotype and exceptional longevity was analyzed in 13 studies [20,22,25,26,27,28,29,30,31,32,33,34,35]. The ID genotype was found to have an OR of 0.86 (95% CI: 0.76–0.97, *p* = 0.01) with no evidence of heterogeneity (0%), indicating a significant negative association between the ID genotype and longevity.

**Figure 4:**
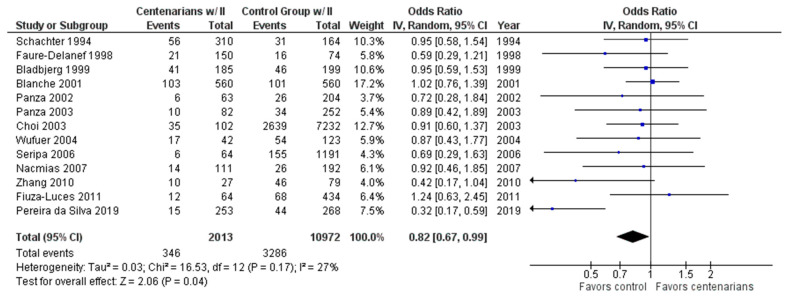
Comparison of II genotype frequency in centenarians and control groups. The ages of centenarians and control groups are as described in Table 1. The *p*-value is indicated by P in the Figure.

As shown in Figure 4, the association between the II genotype and exceptional longevity was analyzed in 13 studies [20,22,25,26,27,28,29,30,31,32,33,34,35]. The II genotype was found to have an OR of 0.81 (95% CI: 0.66–0.98, *p* = 0.03) with mild heterogeneity (28%), indicating a significant negative association between the II genotype and longevity.

### 2.2. Long-Lived Results

Table 2 summarizes the data of long-lived groups. The meta-analysis included 19 studies, with 13 centenarian-only studies, 5 studies that included both nonagenarians and centenarians [23,37,38,39,40], and 1 study with individuals 85 years and older [41], involving a total of 3421 long-lived individuals (85+ years old) and 11,959 controls. Ethnicities of the studied populations were Caucasian (n = 13), Korean (n = 1), Uyghur (n = 1), Han Chinese (n = 2), Russian/Yakut (n = 1), and Peruvian (n = 1).

**Figure 5:**
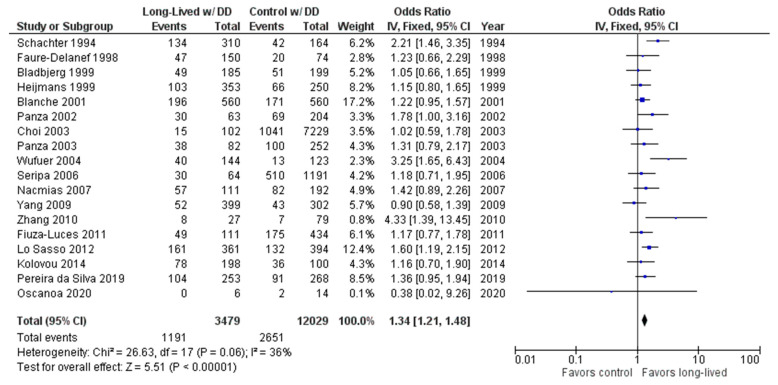
Comparison of DD genotype frequency in long-lived and control groups. The ages of long-lived and control groups are described in Table 1. The *p*-value is indicated by P in the Figure.

The association between the DD genotype and exceptional longevity was analyzed in 18 studies [20,22,23,25,26,27,28,29,30,31,32,33,34,35,38,39,40,41] (Figure 5). The DD genotype was found to have an OR of 1.32 (95% CI: 1.19–1.47, *p* < 0.00001) with mild heterogeneity (30%), indicating a significant positive association between the DD genotype and longevity.

**Figure 6:**
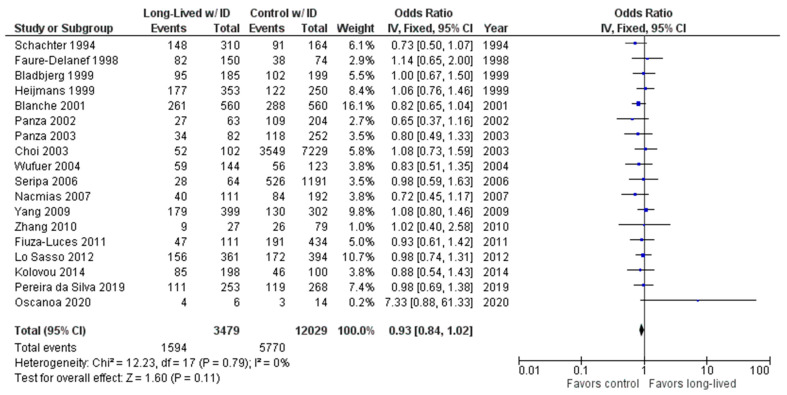
Comparison of ID genotype frequency in long-lived and control groups. The ages of long-lived and control groups are described in Table 1. The *p*-value is indicated by P in the Figure.

As shown in Figure 6, the association between the ID genotype and exceptional longevity was analyzed in 18 studies [20,22,23,25,26,27,28,29,30,31,32,33,34,35,38,39,40,41].

The ID genotype was found to have an OR of 0.92 (95% CI: 0.84–1.02, *p* = 0.11) with no evidence of heterogeneity (0%). There was no significant difference in the frequency of the ID genotype between the long-lived (85+ years old) and control groups.

**Figure 7:**
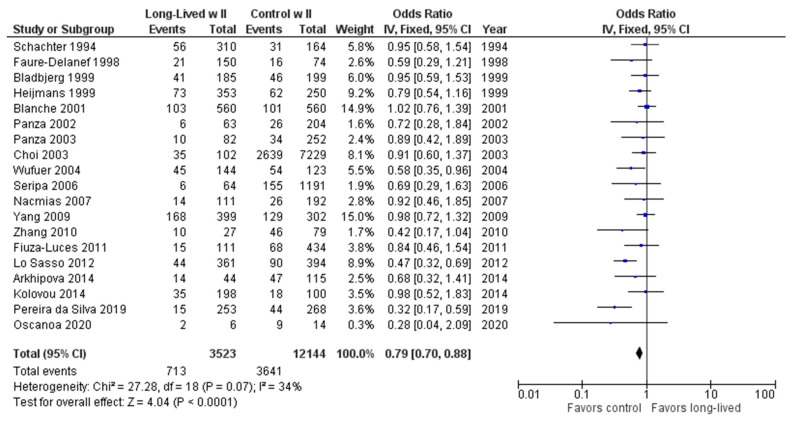
Comparison of II genotype frequency in long-lived and control groups. The ages of long-lived and control groups are described in Table 1. The *p*-value is indicated by P in the Figure.

**Figure 8:**
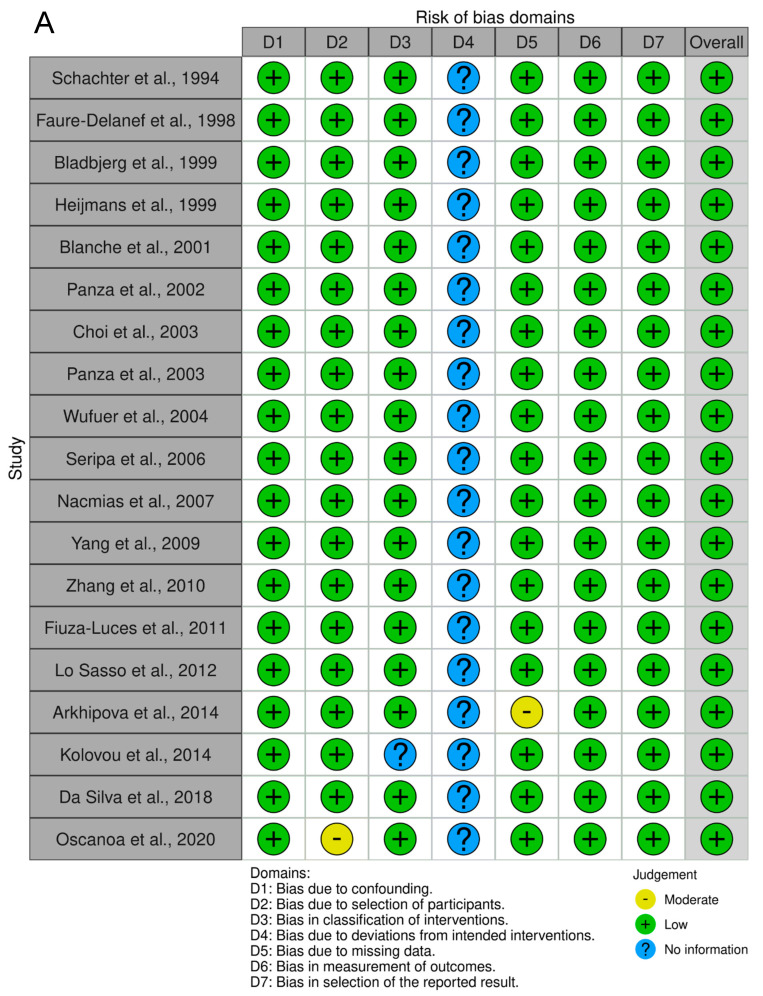

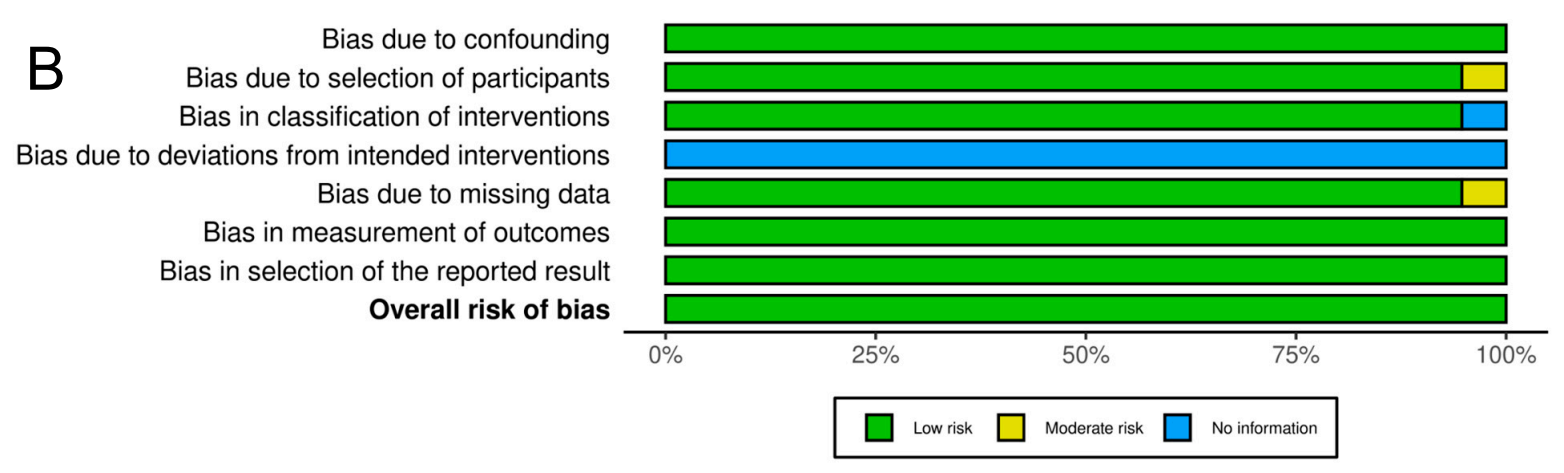
Risk assessment of the studies. (**A**) Traffic light plot showing the assessment of the risk of bias using ROBINS-1. (**B**) Summary plot showing the assessment of the risk of bias using ROBINS-1.

As shown in Figure 7, the association between the II genotype and exceptional longevity was analyzed in 19 studies [20,22,23,25,26,27,28,29,30,31,32,33,34,35,38,39,40,41,42]. Arkhipova et al. (2014) only provided raw frequency data for the II genotype and thus was not included in the DD or ID analyses. The II genotype was found to have an OR of 0.80 (95% CI: 0.71–0.90, *p* = 0.002) with mild heterogeneity (30%), indicating a significant negative association between the II genotype and longevity. The risk of bias of the studies was assessed using ROBINS-1 (method). As shown in Figure 8, the result suggests an overall low risk of bias.

## 3. Discussion

Centenarians are the fastest-growing demographic group of the world’s population, having roughly doubled every decade since 1950 [42]. It has been nearly a decade since the most recent meta-analysis was published in 2013 [21], and additional studies on centenarians and nonagenarians have been conducted that further elucidate the association between *ACE* genotype and longevity. In addition, the use of AI and machine-learning software has aided in the search and screening process to compile even more studies and process the associated data. Our meta-analysis suggests, with increased confidence, that there is a significant positive association of the DD genotype with exceptional longevity across populations of different ethnicities. It has been reported that serum ACE levels correlate in the order of DD > ID > II, in which DD is the highest [11,12,13]. The higher levels of ACE were correlated with increased lifespans, especially more in centenarians than in long-lived controls.

The results present a couple of paradoxes in which the D-allele has been reported as a risk factor for hypertension [14], CVD [16], pneumonia [18], and COVID-19 [19], all of which are major causes of death in older people. However, the DD genotype has been associated with a decreased risk of Alzheimer’s disease, potentially due to ACE’s ability to inhibit amyloid beta aggregation and degrade A beta-(1-40) [43,44,45]. Although the protective effect of the D-allele against Alzheimer’s disease does not fully explain the higher proportion of DD individuals in centenarian cohorts, the D-allele or DD genotype may impart a health risk while also providing a long-term longevity advantage. The middle-life crisis theory of aging [8,46,47,48], in which midlife events are the catalyst for the transition from normal aging to a more advanced pathological state, can be used to explain ACE’s dual functions as an angiotensin-converting enzyme and an amyloid-degrading enzyme [8]; low levels of ACE plasma activity, associated with the II genotype, may initially confer stress resistance before midlife, but will be insufficient to counter the abnormal accumulation of beta-amyloid when it begins and lead to higher mortality rates earlier in the aging process due to AD, whereas DD individuals who have survived midlife comorbidities would now have lower mortality rates from AD. However, this does not fully explain the *ACE* paradox, as Alzheimer’s disease is only one of many diseases that affect older people. As the full role of ACE in the human body has not been determined, the D-allele may have some neuroendocrine or immunomodulatory functions that have yet to be discovered [49] that may further explain *ACE*’s role in longevity. This raises an unanswered key question as to how exceptional longevity is associated with positive risks of age-onset diseases that may increase the risk of mortality. Interestingly, the study of two traits (IP6K3 and IPMK) suggests that gene–gene interactions may be more meaningful than single polymorphisms [50]. Thus, studies of interaction networks are needed [51].

Another paradox is that the inhibition of ACE by mutations or drugs can extend lifespans in model systems from nematodes, fruit flies, and mammals [8,52]. This observation raises awareness that life-extension interventions in model systems may be different from exceptional longevity in humans. Thus, it raises another unanswered key question of whether exceptional longevity in humans shares common underlying mechanisms with life extension in model systems.

Our meta-analysis includes a comprehensive review of studies performed on centenarians, from Schachter’s initial study in 1994 up to the present, for a total of 2054 centenarians. We did not limit the literature search to only studies written in English, thus our data represent all of the current *ACE* centenarian studies in the databases searched. The results corroborated the positive association between the DD genotype and exceptional longevity and the negative association between the II genotype and exceptional longevity that have been noted in previous meta-analyses. A novel finding was that the ID genotype, which had previously shown no significant associations with longevity, was found to have a significant negative association with longevity in the centenarian-only analysis.

It is implicated that the effect of *ACE* on health may differ among different age groups [8]. Thus, we compared the centenarian-only data with the long-lived (85+ years old) data. We found that the distributions of *ACE* DD and II genotypes were consistent across both age groups, with strong associations in the DD genotype with exceptional longevity and with negative associations in the II genotype. Interestingly, the ID genotype had a significant negative association among centenarians but did not show a significant association among long-lived groups. The straightforward interpretations are as follows: (1) The DD genotype is associated with centenarians and the long-lived groups, and the D alleles follow the inheritance pattern of autosomal recessive. (2) The II genotype is negatively associated with longevity in both age groups. (3) The ID genotype shows a weakly negative association with centenarians, but the trend was not significant in the long-lived control. Thus, more detailed studies with a series of ages will be needed.

The limitations of this study are as follows. First, one issue can be summarized as a general limitation of the meta-analysis method. Each search engine lists the papers that are indexed by their criteria, which may mean missing relevant studies. We included three search engines to reduce this risk of missing studies. Second, related to the first limitation is that the criteria to analyze the data rely on the criteria used in each study. For example, control groups of the past studies ranged broadly and were diverse (18–85 years old). There was a lack of detailed age groups to compare the effect of *ACE*. Thus, we were not able to have a series of ages to define age-dependent enrichment of the *ACE* D and I alleles. Third, another limitation is that the vast majority of the centenarian studies on *ACE* used Caucasian populations. Selectively analyzing the studies of Caucasian populations gives the same results as the pooled data, indicating that there were no major differences in associations across ethnicities within this present review. However, there were not enough Asian studies to sufficiently analyze the strength of association within Asian populations only. We also did not find any studies on *ACE* polymorphisms in Hispanic/Latino or Black centenarian cohorts in our literature search. For future meta-analyses, a greater diversity of such studies should be taken to better illuminate the role of *ACE* in aging populations across all different environmental or lifestyle risk factors.

## 4. Materials and Methods

### 4.1. Nomenclature, PRISMA, and Database Search

Due to nomenclature inconsistency among studies, we used I (insertion) and D (deletion) alleles as follows: The genotypes were referred to as homozygous insertion (II), heterozygous insertion/deletion (ID), and homozygous deletion (DD). The I and D polymorphisms were referred to as I/D. We followed the Preferred Reporting Items for Systematic Reviews and Meta-Analyses (PRISMA) [53] and performed a meta-analysis as described [54] with updates described in PRISMA 2020 [55]. We performed a literature search to collect publications on *ACE* I/D polymorphisms in centenarians published before 25 May 2022. A total of 80 studies were obtained from the Human Genomic Aging Resource’s Longevity Map database, Scopus, PubMed, and Google Scholar using the keywords “ACE” OR “angiotensin-converting enzyme” AND “longevity” OR “centenarian” OR “nonagenarian” (subjects 90+ years). Studies of all languages were included. The titles and abstracts of each article were imported to Colandr [24], which uses AI and machine-learning algorithms to determine an article’s relevance based on text-based evidence. Of the 80 studies initially identified, 37 were excluded as duplicates. The remaining 43 articles were then screened in Colandr according to specified inclusion criteria.

### 4.2. Inclusion Criteria

Studies were included if they met all of the following conditions: (1) evaluation of the *ACE* I/D polymorphisms and longevity; (2) case-control study design; (3) study provides sample size, genotype data, odds ratio (OR), and 95% confidence interval (CI) or the data necessary to calculate the results. The exclusion criteria of the studies were as follows: (1) not relevant to *ACE* I/D polymorphisms or longevity; (2) review or meta-analysis; (3) minimum long-lived population age <85 years or unspecified; (4) no control group or maximum control group age >85 years. Data were then separated into centenarian-only and long-lived (85+ years old) sections. Studies were included in the centenarian-only section if the mean population age was >100. In total, we obtained 13 papers that met all our inclusion criteria for centenarians and 19 papers for the long-lived group.

### 4.3. Data Extraction

Data were extracted independently by the author and an assistant, and any discrepancies were settled by consensus. The data extracted included the author’s name, year of publication, ethnicity of the study population, sample size, and genotype numbers in long-lived and control groups. Allele frequencies were calculated if not directly reported. If the study had multiple control groups of varying age ranges, the control group was defined as all individuals ≤85 years of age, and data were extracted accordingly.

### 4.4. Data Synthesis, Statistics, and Assessment of the Risk of Bias

To determine the association between *ACE* genotype and longevity, the odds ratio (OR) with the corresponding 95% confidence interval (CI) was calculated for each genotype (DD, ID, and II). All statistical analyses were performed using The Cochrane Collaboration’s Review Manager (RevMan) Version 5.4 using the random effects statistical model and inverse variance methods to determine the odds ratio, *p*-value, and heterogeneity I2, among others, as described previously [54]. We used a threshold of *p*-value < 0.05 to judge statistical significance. The risk of bias in the studies was evaluated as described previously [54]. The result was visualized with Risk-of-bias VISualization (robvis) ROBINS-1 [56,57]. Bias due to deviations from intended interventions did not apply to this meta-analysis, as participants were categorized by age and could not be blinded to the group they were assigned.

## 5. Conclusions

Our meta-analysis suggests a significant positive association between the *ACE* DD genotype and centenarians and long-lived groups. Although *ACE* polymorphisms are a risk factor for Alzheimer’s disease and age-onset diseases that may contribute to mortality, the *ACE* DD genotype but not the ID genotype was in favor of exceptional longevity over the mortality expected in older people. The DD genotype is associated with a reduced risk of Alzheimer’s disease and increased longevity. It is consistent with an ACE function as an amyloid degradation enzyme, which may be important in exceptional longevity in humans. Additionally, the DD genotype causes a high circulating ACE. In contrast, the DD genotype is also associated with an increased risk of various other diseases. These include hypertension [14], ischemic stroke [15], coronary artery disease [16], left ventricular hypertrophy [17], and pneumonia [18]. Some of these diseases, including hypertension and cardiovascular conditions (stroke and coronary artery disease), are associated with the risks of COVID-19 mortality [54]. The DD genotype is associated with higher COVID-19 mortality [19]. This may imply disease interactions mediated by the D allele. The underlying mechanisms of human longevity remain unclear, especially when considering the D-allele’s role as a risk factor for many age-related comorbidities. Further studies on nonagenarians and centenarians of different backgrounds may provide better insight into the role of *ACE* as a longevity gene.

## Figures and Tables

**Table 1:** Characteristics of studies on *ACE* I/D polymorphisms in centenarians. A total of 13 studies were included that looked at the frequency of different *ACE* I/D polymorphisms in centenarians.

	Year	Centenarian		Control		Ethnicity	Ref
		# of Subjects	Age (Years) Mean ± SD	# of Subjects	Age (Years) Range		
Schachter et al.	1994	310	100.71 ± 0.13	164	20–70	Caucasian	[20]
Faure-Delanef et al.	1998	150	100.6	74	20–70	Caucasian	[25]
Bladbjerg et al.	1999	185	>100	199	20–64	Caucasian	[26]
Blanche et al.	2001	560	103.1	560	18–70	Caucasian	[27]
Panza et al.	2002	63	100.3 ± 1.9	204	19–80	Caucasian	[28]
Choi et al.	2003	102	102.4 ± 2.6	7229	20–85	Korean	[22]
Panza et al.	2003	82	100 ± 2	252	19–70	Caucasian	[29]
Wufuer et al.	2004	42	103.0 ± 3.0	53	65–70	Uyghur	[30]
Seripa et al.	2006	64	100.28 ± 1.86	1191	22–59	Caucasian	[31]
Nacmias et al.	2007	111	102.4 ± 2.6	192	84 ± 18.2	Caucasian	[32]
Zhang et al.	2010	27	>100	79	60–70	Han Chinese	[33]
Fiuza-Luces et al.	2011	65	101.7 ± 1.8	283	21.2 ± 2.0	Caucasian	[34]
Pereira da Silva et al.	2018	253	100.26 ± 1.98	268	67.51 ± 3.25	Caucasian	[35]

**Table 2:** Characteristics of studies on *ACE* I/D polymorphisms in long-lived individuals (85+ years old).

	Year	Long-Lived		Control		Ethnicity	Ref
		# of Subjects	Age (Years) Mean ± SD	# of Subjects	Age (Years) Range/Mean ± SD		
Schachter et al.	1994	310	100.71 ± 0.13	164	20–70	Caucasian	[20]
Faure-Delanef et al.	1998	150	100.6	74	20–70	Caucasian	[25]
Bladbjerg et al.	1999	185	>100	199	20–64	Caucasian	[26]
Heijmans et al.	1999	353	85–100	250	18–40	Caucasian	[41]
Blanche et al.	2001	560	103.1	560	18–70	Caucasian	[27]
Panza et al.	2002	63	100.3 ± 1.9	204	19–80	Caucasian	[28]
Choi et al.	2003	102	102.4 ± 2.6	7229	20–85	Korean	[22]
Panza et al.	2003	82	100 ± 2	252	19–70	Caucasian	[29]
Wufuer et al.	2004	42	103.0 ± 3.0	53	65–70	Uyghur	[30]
Seripa et al.	2006	64	100.28 ± 1.86	1191	22–59	Caucasian	[31]
Nacmias et al.	2007	111	102.4 ± 2.6	192	84 ± 18.2	Caucasian	[32]
Yang et al.	2009	399	>90	302	<60	Han Chinese	[40]
Zhang et al.	2010	27	>100	79	60–70	Han Chinese	[33]
Fiuza-Luces et al.	2011	65	101.7 ± 1.8	283	21.2 ± 2.0	Caucasian	[34]
Lo Sasso et al.	2012	361	97	394	39	Caucasian	[39]
Arkhipova et al.	2014	44	>90	115	60–74	Russian, Yakut	[37]
Kolovou et al.	2014	198	≥90	105	18–79	Caucasian	[38]
Pereira da Silva et al.	2018	253	100.26 ± 1.98	268	67.51 ± 3.25	Caucasian	[35]
Oscanoa et al.	2020	6	>85	14	<65	Peruvian	[23]

## Data Availability

The data supporting the reported results have been made available within the manuscript.

## Abbreviations

ACE	angiotensin-converting enzyme
I	insertion
D	deletion
CVD	cardiovascular disease
COVID-19	coronavirus 2019
OR	odds ratio
CI	confidence interval
